# MITF Promotes Cell Growth, Migration and Invasion in Clear Cell Renal Cell Carcinoma by Activating the RhoA/YAP Signal Pathway

**DOI:** 10.3390/cancers13122920

**Published:** 2021-06-11

**Authors:** Nayoung Kim, Solbi Kim, Myung-Won Lee, Heung-Jin Jeon, Hyewon Ryu, Jin-Man Kim, Hyo-Jin Lee

**Affiliations:** 1 Department of Medical Science, College of Medicine, Chungnam National University, Daejeon 34134, Korea; kimny80@nate.com (N.K.); kimsb2662@naver.com (S.K.); 2 Department of Internal Medicine, College of Medicine, Chungnam National University, Daejeon 34134, Korea; iyoo23@cnuh.co.kr (M.-W.L.); ryhw001@naver.com (H.R.); 3 Infection Control Convergence Research Center, College of Medicine, Chungnam National University, Daejeon 34134, Korea; livinglogos@cnu.ac.kr; 4 Department of Pathology, College of Medicine, Chungnam National University, Daejeon 34134, Korea; jinmank@cnu.ac.kr

**Keywords:** renal cell carcinoma, MITF, RhoA, YAP, proliferation, cell cycle, migration, invasion

## Abstract

**Simple Summary:**

Microphthalmia-associated transcription factor (MITF) has been reported to play a role in the progression of melanoma and other cancer types. However, the biological role of MITF in clear cell renal cell carcinoma (ccRCC) is largely unknown. In this study, we elucidate the role of MITF in the progression of ccRCC. MITF- and MITF-mediated signaling pathways were investigated in ccRCC cell through MITF knockdown as well as overexpression of MITF in vitro and in vivo. MITF contributed to cell proliferation, migration, invasion and tumor growth in ccRCC through activation of the RhoA/YAP signaling pathways. This study suggests that MITF has potential as a therapeutic target in ccRCC.

**Abstract:**

Microphthalmia-associated transcription factor (MITF) is a basic helix-loop-helix leucine zipper transcription factor involved in the lineage-specific regulation of melanocytes, osteoclasts and mast cells. MITF is also involved in the progression of melanomas and other carcinomas, including the liver, pancreas and lung. However, the role of MITF in clear cell renal cell carcinoma (ccRCC) is largely unknown. This study investigates the functional role of MITF in cancer and the molecular mechanism underlying disease progression in ccRCC. MITF knockdown inhibited cell proliferation and shifted the cell cycle in ccRCC cells. In addition, MITF knockdown reduced wound healing, cell migration and invasion compared with the controls. Conversely, MITF overexpression in SN12C and SNU482 cells increased cell migration and invasion. Overexpression of MITF activated the RhoA/YAP signaling pathway, which regulates cell proliferation and invasion, and increased YAP signaling promoted cell cycle-related protein expression. Additionally, tumor formation was impaired by MITF knockdown and enhanced by MITF overexpression in vivo. In summary, MITF expression was associated with aggressive tumor behavior, and increased the migratory and invasive capabilities of ccRCC cells. These effects were reversed by MITF suppression. These results suggest that MITF is a potential therapeutic target for the treatment of ccRCC.

## 1. Introduction

Renal cell carcinoma (RCC) is the most common form of kidney cancer; it typically originates from renal tubular epithelial cells and accounts for about 2–3% of all malignancies in adults [1]. Clear cell RCC (ccRCC) is a major subtype of RCC, accounting for approximately 70–80% of cases. The survival rate of ccRCC is poorer than those of other types of RCC (e.g., papillary, chromophobe) because of its aggressive clinical course and resistance to chemotherapy and radiotherapy [2,3,4]. About 30% of ccRCC patients eventually develop metastasis, and less than 10% of these patients survive for 5 years or more after diagnosis [5]. This poor prognosis underlies the need for a better understanding of the mechanism of metastatic ccRCC, to enable development of potent therapeutics.

The Hippo signaling pathway is a key regulator of tumor cell proliferation and invasion in various types of cancers, including esophageal squamous cell carcinoma [6], breast cancer [7], hepatocellular carcinoma [8] and cervical cancer [9]. Yes-associated protein (YAP) is a key effector of the Hippo pathway. Under normal conditions, YAP forms a complex with TEAD proteins [10] and is translocated to the nucleus to promote cell growth. Activation of the Hippo pathway causes YAP phosphorylation, leading to cytoplasmic retention and non-proteolytic ubiquitination or proteolytic degradation of YAP [11,12]. The Hippo–YAP pathway is directly involved in cancer development and regulates many genes that function in survival and the epithelial–mesenchymal transition (EMT), among other processes [13,14]. This makes YAP inhibition an attractive target for cancer prevention and treatment [15,16].

Microphthalmia-associated transcription factor (MITF), which contains a basic helix-loop helix and leucine zipper (bHLH-LZ) structure, has been established as a major regulator of melanocyte development [17,18]. It regulates key genes responsible for melanocyte cell development, differentiation, survival and pigment production, as well as cell-cycle regulation [19]. However, subsequent studies have suggested that MITF has multiple effects in cancers such as hepatocellular carcinoma [20], pancreatic cancer [21], lung cancer [22] and papillary RCC [23]. Nevertheless, the role of MITF signaling in ccRCC is still unclear. Thus, we investigated the biological function of MITF and its effect on tumor growth and cancer progression in ccRCC.

## 2. Materials and Methods

### 2.1. Cell Lines and Cell Culture

The human ccRCC cell lines SNU1272, Caki-2, SN12C and SNU482 were purchased from the Korean Cell Line Bank (Seoul, Korea)**.** SNU1272 and SNU482 were maintained in RPMI1640 (Welgene, Daejeon, Korea), supplemented with 10% fetal bovine serum (FBS) and 1% penicillin/streptomycin (P/S). Caki-2 and SN12C were maintained in Dulbecco’s Modified Eagle’s Medium (DMEM; Welgene, Daejeon, Korea) supplemented with 10% FBS and 1% P/S, respectively. Cells were cultured at 37 °C under 5% CO_2_ and 95% relative humidity.

### 2.2. Reagents and Antibodies

Antibodies against MITF (12590; Cell Signaling Technology, Danvers, MA, USA), GAPDH (sc-25778; Santa Cruz Biotechnology, Santa Cruz, CA, USA), p-21 (Santa Cruz Biotechnology; sc-6246), CDK-2 (Cell Signaling Technology; 2546), CDK1 (Santa Cruz Biotechnology; sc-136014), cyclin A (Santa Cruz Biotechnology; sc-271682), cyclin B1 (Santa Cruz Biotechnology; sc-245), cyclin E (Santa Cruz Biotechnology; sc-377100), phosphor-FAK (Cell Signaling Technology; 3283), FAK (Cell Signaling Technology; 3285), F-actin (ab205; Abcam, Cambridge, UK), phospho-YAP (Cell Signaling Technology; 4911), YAP (Cell Signaling Technology; 4912), lamin B (Santa Cruz Biotechnology; sc-6216), Rac1–3 (Cell Signaling Technology; 2465), Cdc42 (Cell Signaling Technology; 2466), phospho-Rac1/Cdc42 (Cell Signaling Technology; 2461), RhoA (Cell Signaling Technology; 2117 or Santa Cruz Biotechnology; sc-418) and CRIK (Santa Cruz Biotechnology; sc-390437) were used in the Western blot and immunofluorescence analyses. CN03 was purchased from Cytoskeleton (Denver, CO, USA).

### 2.3. MITF Knockdown in RCC Cell Lines

MITF knockdown in ccRCC cells was achieved via lentivirus-mediated transduction of MITF siRNA into a pLKO.1-puro lentiviral vector (Clontech, Mountain View, CA, USA). For stable transfection, the lentiviral vector was co-transfected into HEK-293T (Clontech) cells with virus mix (Sigma, St. Louis, MO, USA) using Lipofectamine 3000 (Invitrogen, Carlsbad, CA, USA), according to the manufacturer’s instructions. The virus was harvested from the supernatant and concentrated with Lenti-X-Concentrator (Clontech). The virus was added to SNU1272 and Caki-2 cells along with 5 µg/mL polybrene (Santa Cruz Biotechnology). After 20 h, the medium was removed and replaced with fresh medium containing 3 µg/mL puromycin (Sigma). Puromycin-resistant clones were identified by culture for 2 weeks in the presence of puromycin. MITF-KD expression was analyzed by Western blot and RT-PCR analysis.

### 2.4. Overexpression of MITF in RCC Cell Lines

MITF overexpression in ccRCC cells was achieved using pEGFP-N1-MITF plasmid (Clontech). pEGFP-N1-MITF plasmid was transfected into SN12C and SNU482 cells using Lipofectamine 3000 (Invitrogen) according to the manufacturer’s instructions. After 48 h, the medium was removed and replaced with fresh medium containing 1 mg/mL G418 (Sigma). G418-resistant clones were identified by culture for 2 weeks in the presence of G418. MITF levels were analyzed by Western blot.

### 2.5. Transient Transfection

pDK-flag-YAP-WT, pDK-flag-YAP-2SA and control vector plasmids were provided by Prof. Chung (Chungnam National University, Daejeon, Korea). Transfection of the different DNA constructs was performed using Lipofectamine 2000 (Invitrogen) according to the manufacturer’s instructions. Further assays were conducted after incubating the transiently transfected cells for 48 h.

### 2.6. In Vitro Cell Proliferation Assay

Cell proliferation was measured by direct cell counting. Briefly, the cells were seeded in triplicate in a 60 mm dish at a density of 2 × 10^5^ cells. After incubation for 1–3 days, the cells were harvested and counted using the trypan blue exclusion test.

### 2.7. Anchorage-Independent Growth (Anoikis) Assay

The anoikis assay was performed using an ultra-low attachment plate (Corning Inc., Corning, NY, USA). Briefly, 1 × 10^3^ cells were seeded in the plate and, after incubation for 14 days, were observed under a microscope.

### 2.8. Clonogenic Assay

For the clonogenic assay, 1 × 10^3^ cells were seeded in a 6-well plate. Once the appropriate colony size had formed, the plates were rinsed with PBS three times and fixed with 10% formalin overnight at 4 °C. Colonies were then stained with 0.1% crystal violet at room temperature for 1 h and observed under a microscope. To determine the relative number of colonies, 70% alcohol was used to elute the crystal violet, and the absorbance was detected at 595 nm using a spectrophotometer.

### 2.9. Migration and Invasion Assays

Chemotaxis and invasion of ccRCC cells were achieved using an 8 µm pore size Transwell chamber (Corning Costar, Cambridge, MA, USA). Migration and invasion assays were performed using our previously reported protocols [24]. Briefly, the lower surface of the Transwell was coated with 10 µg of gelatin for the chemotaxis assay, while the upper side was coated with 25 µg (0.5 μg/μL) reconstituted basement membrane substance only for the invasion assay (Matrigel; BD Biosciences, Franklin Lakes, NJ, USA). Fresh medium containing 10% FBS was placed in the lower chamber as a chemoattractant. ccRCC cells were incubated for 24 h in medium containing 1% FBS, trypsinized, and suspended at a final concentration of 1 × 105 cells/mL in medium containing 0% FBS. Then, 100 μL of the cell suspension was loaded into each of the upper wells, and the chamber was incubated at 37 °C for 24 h (migration) or 48 h (invasion). Cells were fixed and stained with 0.1% crystal violet staining. Chemotaxis activity was quantified by counting the cells that migrated to the lower side of the filter with an optical microscope. Five random fields were counted for each assay.

### 2.10. Western Blot Analysis

Western blotting was performed using our previously reported protocols [24]. Briefly, cells were lysed in RIPA buffer with protease inhibitor cocktail (Sigma) and phosphatase inhibitor cocktail (Roche, Basel, Switzerland). Cell lysates were subjected to SDS-PAGE and then transferred to polyvinylidene fluoride (PVDF) membranes (Pall Corp., Port Washington, NY, USA). The membranes were incubated with the indicated primary antibodies, followed by 1 h incubation with horseradish peroxidase-conjugated secondary antibodies (Cell Signaling Technology). The immunoreactive polypeptides were visualized using a chemiluminescent substrate (Bio-Rad, Hercules, CA, USA; and Thermo, Waltham, MA, USA). Protein band intensities were measured using the Image J software (ver. 1.52v, National Institutes of Health, Bethesda, MD, USA). Original western blots can be found at Figure S7.

### 2.11. Wound Healing Assay

Wound healing assays were performed using specific chambers (Ibidi, Munich, Germany) in accordance with our previously reported protocols [24]. First, 70 μL of cell suspension was seeded at a density of 5 × 10^4^ cells on each side of an Ibidi high 35 mm μ-dish with culture inserts, for live cell analysis. After culturing cells for 24 h, the culture inserts were removed and cells were incubated with fresh culture medium. Cells were monitored over a 16 h period.

### 2.12. Immunofluorescence

Immunofluorescence was performed using our previously reported protocols [24]. Briefly, cells were attached to a Chamber Slide^TM^ (Lab-TekII). After starvation for 6 h in serum-free medium, cells were washed once in phosphate-buffered saline (PBS), fixed in 3.7% formaldehyde (FA) for 10 min at 37 °C, permeabilized with 0.5% Triton X-100 for 20 min at room temperature, washed in PBS, and then blocked in 3% chicken serum albumin (CSA) in PBS for 30 min at room temperature. To visualize MITF, YAP and phalloidin, the cells were stained with anti-MITF, anti-YAP or phalloidin, and fluorescein isothiocyanate-labeled (Sigma; P5282) overnight at 4 °C. Cells were then washed in PBS followed by treatment with secondary antibody for 2 h at 37 °C. Finally, the cells were washed with PBS, their nuclei were stained with DAPI (CA94010; Vector Laboratories, Burlingame, CA, USA), and coverslips were mounted on the slide.

### 2.13. Cell Cycle Analysis

For cell cycle analysis, 1 × 10^5^ cells were seeded in a 6-well plate. After two washes with ice-cold PBS, cells were fixed in 70% ice-cold ethanol overnight at 4 °C. Cells were washed again with ice-cold PBS and incubated with FxCycle™ PI/RNase Staining Solution (Invitrogen) according to the manufacturer’s instructions. Fluorescence was analyzed using a flow cytometer (Beckman Coulter, Brea, CA, USA) and the Kaluza analysis program (version 1.2; Beckman Coulter).

### 2.14. Active RhoA/Rac1/Cdc42 Pulldown Assay

RhoA, Rac1 and Cdc42 activities in cells were measured using the Rac1/Cdc42 activation Assay Kit and Rho assay reagent (Millipore, Burlington, MA, USA) according to the manufacturer’s instructions. Cells were seeded in 100 mm dishes, cultured to 60–80% confluence, and serum-starved for 6 h. The cells were washed with ice-cold PBS and lysed with cell lysis buffer. Lysates were harvested and incubated with 10–20 μg Rhotekin-RBD or PAK-PBD protein agarose beads for 1 h at 4 °C. Pellets were washed four times and subjected to Western blot analysis using anti-RhoA, anti-Rac1 and Cdc42 antibodies.

### 2.15. Xenograft Tumors in Nude Mice

Balb/c-nude mice (4-week-old females) were purchased from DooYeol Biotech (Seoul, Korea) and maintained in a pathogen-free environment. We injected 1 × 10^6^ cells subcutaneously into left and right mouse flanks with SNU1272-shCtrl, SNU1272–shMITF, SN12C-Ctrl or SN12C-MITF cells. The tumor weight and diameter were measured after successful inoculation of stably transfected cells. The tumor volume (V) was calculated using the following formula: V = 0.5 × L × W × W (L, length; W, width). Animals were euthanized 60 days after inoculation and the tumor masses were removed by microsurgical dissection. Specimens were formalin-fixed, paraffin-embedded, serially sectioned on 200 µm sections, and stained with hematoxylin and eosin (H&E). All animals received humane care according to institutional guidelines, and all experiments were approved by the Institutional Review Board of CNUH (approval number: CNUH-017-A0035). 

### 2.16. Statistical Analysis

All experiments were performed in at least triplicate. Student’s *t*-test was used to analyze differences between two groups using Microsoft Excel 2016 software (Microsoft Corp., Redmond, WA, USA). One-way analysis of variance (ANOVA) was used to analyze multiple comparisons using GraphPad Prism software (version 5.0; GraphPad Software Inc., La Jolla, CA, USA). A *p*-value less than 0.05 was considered statistically significant.

## 3. Results

### 3.1. Knockdown of MITF Inhibits Cell Growth by Cell Cycle Shift

**Figure 1:**
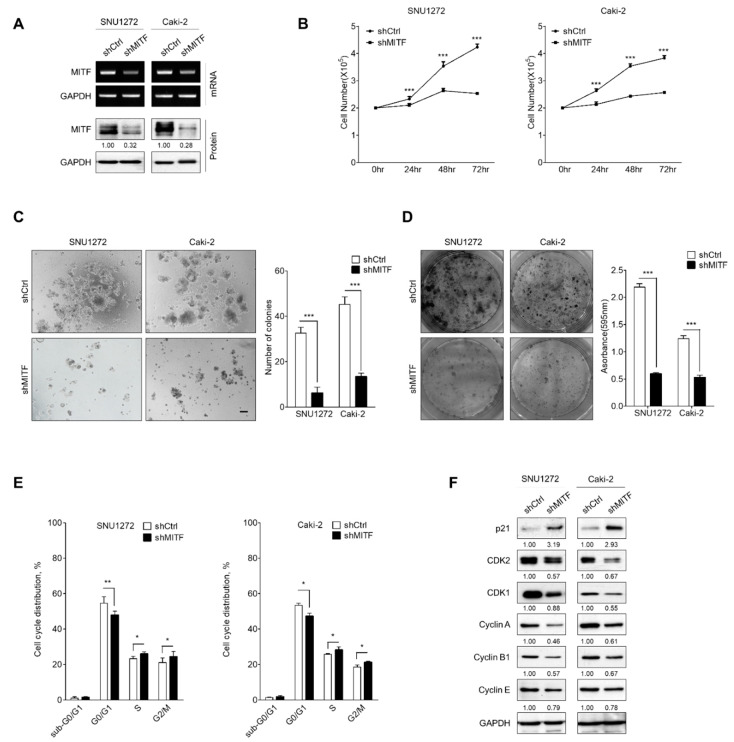
MITF knockdown suppresses cell proliferation and cell cycle progression. (**A**) Western blot analysis and RT-PCR of MITF in MITF-knockdown and shCtrl cells. Whole areas were measured using Image J software (*n* = 3). Uncropped blots are shown in Figure S7. (**B**) Cell proliferation rates by cell counting assay in MITF-knockdown and shCtrl cells. Data are presented as the means ± standard deviation and were evaluated using Student’s *t*-test (*n* = 3). (**C**) The anoikis assay was performed on an ultra-low attachment cell-culture plate. Single-cell suspensions grew into large spheroids. Data are presented as the means ± standard deviation and were evaluated using Student’s *t*-test (*n* = 5). Scale bar, 100 μm. (**D**) The clonogenic assay was performed on a 6-well culture plate. Crystal violet-stained cells were dissolved in 70% alcohol, and absorbance at 595 nm was measured using a spectrophotometer. The data are presented as the mean ± standard deviation and were evaluated using Student’s *t*-test (*n* = 3). (**E**) Cell cycle analysis by flow cytometry and the percentage of cells in sub-G0/G1, G0/G1, S and G2/M phases are annotated in each column. Data are presented as the means ± standard deviation and were evaluated using Student’s *t*-test (*n* = 4). (**F**) Western blot analysis showing expression of cell cycle-related proteins. Whole areas were measured using Image J software (*n* = 3). Uncropped blots are shown in Figure S7. *, *p* < 0.05; **, *p* < 0.01; ***, *p* < 0.001.

To investigate how MITF regulates tumor progression in ccRCC, we established two ccRCC cell lines (SNU1272 and Caki-2) with stable MITF knockdown (pLKO.1-shCtrl or pLKO.1-shMITF). We confirmed knockdown of MITF by reverse transcription polymerase chain reaction (RT-PCR), Western blot analysis and immunofluorescence staining (Figure 1A and Figure S1). Suppressing MITF expression effectively attenuated ccRCC cellular growth, as determined by cell counting, anchorage-independent growth assay and colony formation assay (Figure 1B–D). The proportions of cells in S and G2/M phases of the cell cycle were increased after knockdown, while the proportion in the G0/G1 phase was decreased in both MITF-knockdown cell lines (*p* < 0.001) (Figure 1E and Figure S2). Additionally, Western blot analysis revealed that the cell cycle regulatory proteins CDK1, CDK2, cyclin A, cyclin B1 and cyclin E were downregulated after MITF knockdown, whereas Cdk inhibitor p21 was upregulated (Figure 1F). These findings suggest that suppressing MITF significantly inhibited cell growth via a cell cycle shift in the ccRCC.

### 3.2. Knockdown of MITF Suppresses Cell Migration and Invasion In Vitro

**Figure 2:**
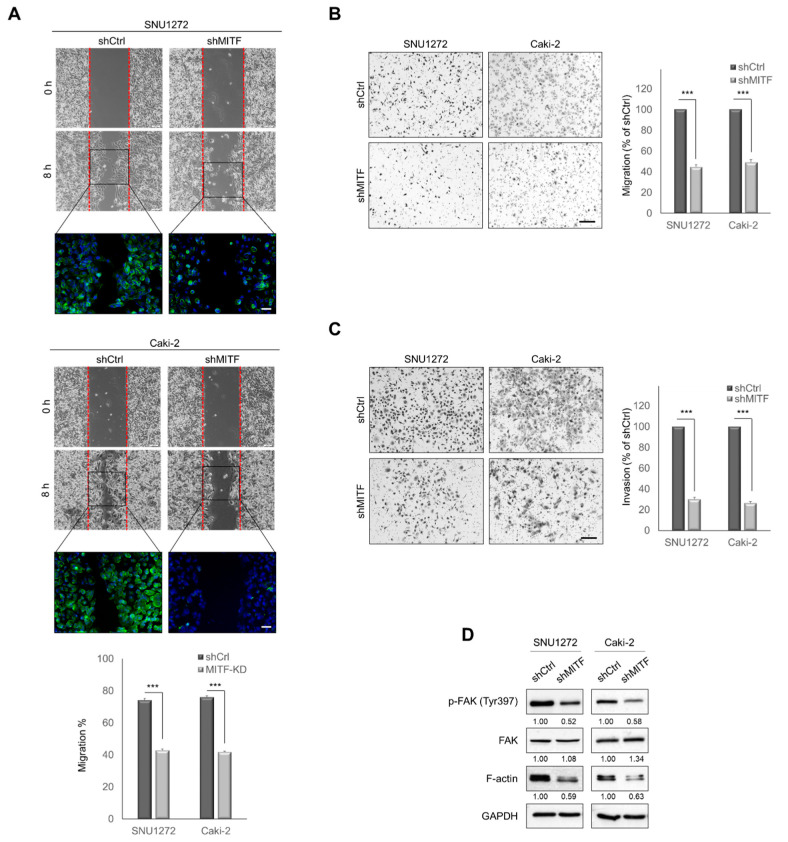
MITF-knockdown suppresses cell migration and invasion in vitro. (**A**) Wound-healing assay in MITF-knockdown and shCtrl cells at 0 and 8 h. Scale bars, 100 μm. Square box indicates FITC-conjugated phalloidin staining by immunofluorescence microscopy (×400). Data are presented as the means ± standard deviation and were evaluated using Student’s *t*-test (*n* = 3). Scale bars, 20 μm. (**B**,**C**) Transwell migration and invasion assays. The number of cells in five randomly chosen fields was counted. Data are presented as the means ± standard deviation and were evaluated using Student’s *t*-test (*n* = 3). Scale bar, 200 μm. (**D**) Western blot analysis of phosphorylated FAK and F-actin expression in MITF-knockdown cells. Whole areas were measured using Image J software (*n* = 3). Uncropped blots are shown in Figure S7. ***, *p* < 0.001.

MITF-knockdown cells demonstrated significantly impaired cell motility in a wound healing assay (Figure 2A). While shCtrl cells nearly closed 0.5 mm wide gaps within 8 h, MITF-knockdown cells significantly inhibited wound closure and produced fewer stress fibers compared with the control cells (Figure 2A and Figure S3). In addition, MITF-knockdown cells showed significantly reduced migration and invasion capability compared with the shCtrl cells (Figure 2B, C). As shown in Figure 2D, MITF-knockdown decreased the phosphorylation of FAK and F-actin expression. These results suggest that knockdown of MITF suppresses cell motility, migration and invasion.

### 3.3. Knockdown of MITF Inhibits YAP Nuclear Translocation

**Figure 3:**
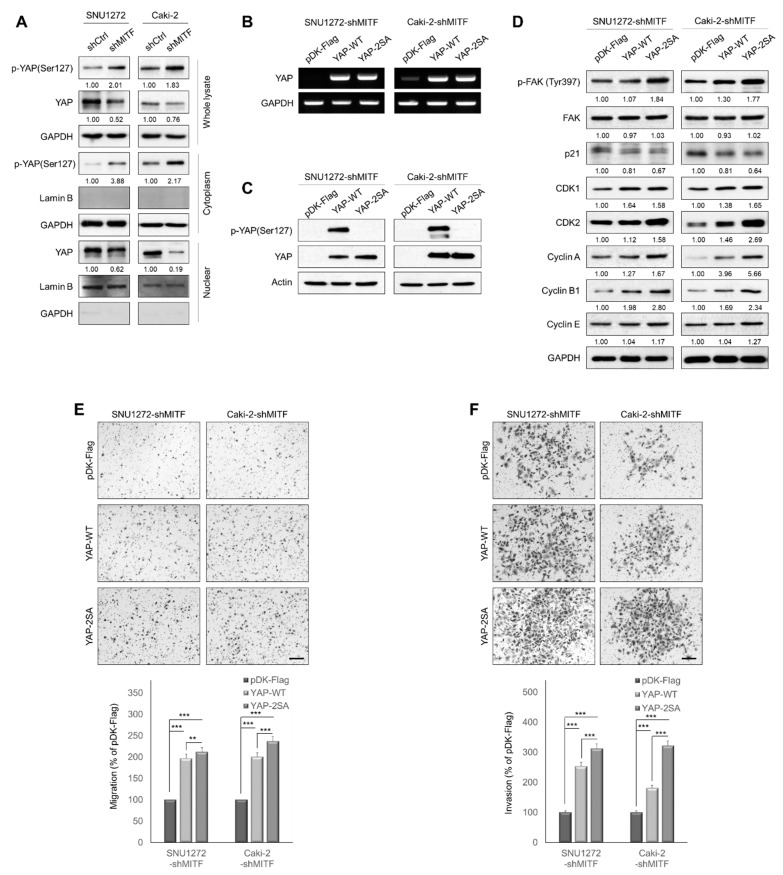
MITF-knockdown inhibits YAP nuclear translocation. (**A**) Western blot analysis of nuclear and cytoplasmic YAP and phosphorylated YAP in MITF-knockdown cells compared with shCtrl cells. Whole areas were measured using Image J software (*n* = 3). Uncropped blots are shown in Figure S7. (**B**,**C**) YAP overexpression levels were determined by Western blot analysis and RT-PCR in MITF-knockdown cell lines. Uncropped blots are shown in Figure S7. (**D**) Western blot analysis of cell cycle-related proteins. Whole areas were measured using Image J software (*n* = 3). Uncropped blots are shown in Figure S7. (**E**,**F**) Transwell migration and invasion assay showing that YAP overexpression restored cell migration and invasion suppressed by MITF knockdown. The number of cells in five randomly chosen fields was counted. Data are presented as the means ± standard deviation by one-way ANOVA (*n* = 3). Scale bar, 200 μm. **, *p* < 0.01; ***, *p* < 0.001.

Next, we investigated the mechanism by which MITF contributed to ccRCC progression. We screened several signaling molecules related to cancer biology using ccRCC cell lines. As depicted in Figure S4, we observed that MITF knockdown decreased YAP and increased phospho-YAP. We therefore assumed that MITF would influence cancer progression through Hippo-YAP signaling axis. The Hippo-YAP signaling pathway is a key regulator of cell proliferation and metastasis [10]. To determine whether MITF regulates YAP expression, we performed Western blot analyses on fractionated nuclear and cytoplasmic proteins in MITF-knockdown cells. MITF knockdown decreased nuclear YAP protein and increased phosphorylated YAP in the cytoplasmic fraction (Figure 3A). Next, we examined the effects of YAP overexpression in MITF-knockdown cells. We assessed mRNA and protein levels (Figure 3B, C), and performed immunofluorescence staining on cells overexpressing active YAP S127/381A (YAP-2SA) and wild-type cells (YAP-WT) (Figure S5). YAP overexpression increased CDK1, CDK2, cyclin A, cyclin B1 and cyclin E protein levels, as well as FAK phosphorylation, and slightly decreased the p21 level (Figure 3D). YAP overexpression increased cell migration and invasion, which was suppressed by knocking down MITF (Figure 3E,F). Taken together, these findings indicate that YAP plays a crucial role in the MITF-induced cell motility and invasiveness of ccRCC cells.

### 3.4. Knockdown of MITF Suppresses RhoA Activation

**Figure 4:**
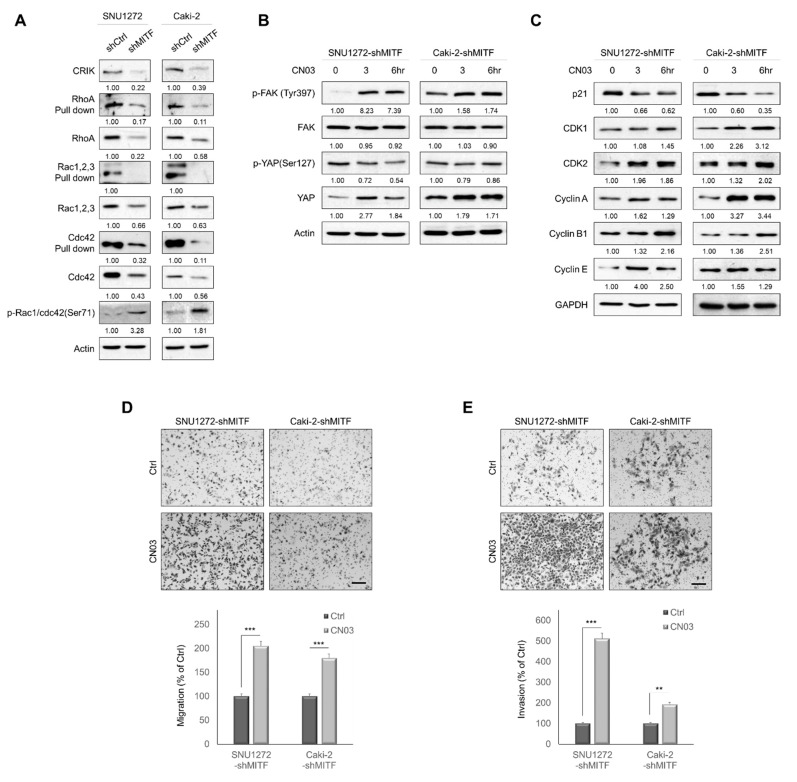
MITF knockdown suppresses RhoA activation. (**A**) Pull-down assay of Rho family proteins shows that knockdown of MITF inhibited activation of RhoA, Rac1–3 and Cdc42. Whole areas were measured using Image J software (*n* = 3). Uncropped blots are shown in Figure S7. (**B**) MITF-knockdown cells were treated with 1 μg/mL of CN03 for 3 or 6 h. Western blot analysis showed that CN03 effectively increased the expression of phospho-FAK and YAP. Whole areas were measured using Image J software (*n* = 3). Uncropped blots are shown in Figure S7. (**C**) Western blot analysis showing expression of cell cycle-related proteins. Whole areas were measured using Image J software (*n* = 3). Uncropped blots are shown in Figure S7. (**D**,**E**) Transwell migration and invasion assay showing that CN03 promoted MITF knockdown-induced cell migration and invasion. The number of cells in five randomly chosen fields was counted. Data are presented as the means ± standard deviation and were evaluated using Student’s *t*-test (*n* = 3). Scale bar, 200 μm. **, *p* < 0.01; ***, *p* < 0.001.

RhoA is known to induce transcriptional gene programs that activate YAP through G-protein-coupled receptors (GPCRs) [25]. Citron Rho-interacting serine/threonine kinase (CRIK) is a downstream substrate of the Rho family of GTPase proteins [26]. We investigated the effect of MITF-knockdown on CRIK levels and GTPase expression in ccRCC cells. Members of the GTPase family (RhoA, Rac 1,2,3 and Cdc42), as well as the downstream intermediate CRIK, are downregulated in MITF-knockdown cells (Figure 4A). CN03, a known RhoA activator, was added to MITF-knockdown cells to determine the impact of RhoA expression. CN03 treatment significantly increased the phosphorylation of FAK. Although YAP expression levels were significantly increased in treated cells, YAP phosphorylation was decreased (Figure 4B). In addition, CDK1, CDK2, cyclin A, cyclin B1 and cyclin E levels were increased by RhoA activation in both cell lines, whereas the p21 level was decreased (Figure 4C). Furthermore, activation of RhoA increased cell migration and invasion, reversing the effect of MITF knockdown (Figure 4D,E). Increased activation of RhoA in MITF-knockdown cell lines induced cell motility and invasiveness of ccRCC cells.

### 3.5. MITF Overexpression Promoted Cell Migration and Invasion by Upregulating RhoA/YAP Signaling

**Figure 5:**
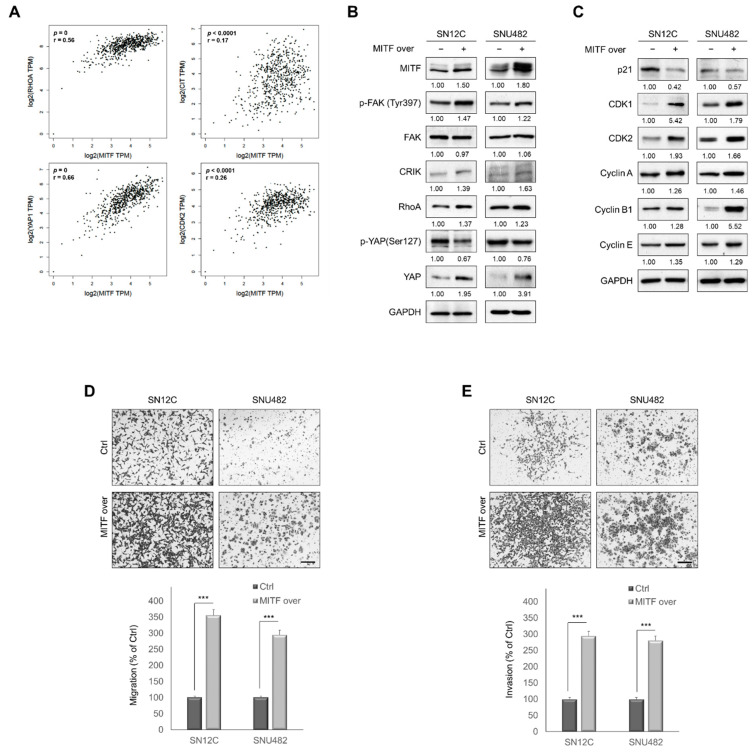
MITF overexpression promotes cell migration and invasion by upregulating RhoA/YAP signaling. (**A**) The four panels (clockwise from top left) show correlation of MITF vs. RhoA, CRIK, CDK2 and YAP1 expression in the GEPIA kidney ccRCC dataset. (**B**) Western blot analysis showing increased expression of phosphorylated FAK, CRIK, RhoA and YAP in MITF-overexpressing cells. Whole areas were measured using Image J software (*n* = 3). Uncropped blots are shown in Figure S7. (**C**) Western blot analysis of cell cycle-related proteins. Whole areas were measured using Image J software (*n* = 3). Uncropped blots are shown in Figure S7. (**D**,**E**) Transwell migration and invasion assay showing that MITF overexpression promoted cell migration and invasion. The number of cells in five randomly chosen fields was counted. Data are presented as the means ± standard deviation and were evaluated using Student’s *t*-test (*n* = 3). Scale bar, 200 μm. ***, *p* < 0.001.

We performed a correlation analysis between MITF and RhoA/YAP expression levels. Figure 5A shows that there is a positive correlation between MITF expression and that of RhoA, CRIK, CDK2 and YAP. To clarify that MITF increases the migration and invasion of ccRCC cells through regulation of RhoA/YAP signaling, we created MITF-overexpressing cell lines transfected with pEGFP-N1 plasmid in SN12C and SNU482 cells (Figure 5B). As shown in Figure 5B, MITF overexpression increased CRIK and RhoA levels, as well as FAK phosphorylation, and decreased YAP phosphorylation. Protein levels of the regulatory proteins CDK1, CDK2, cyclin A, cyclin B1 and cyclin E were significantly increased by MITF overexpression in both cell lines, while p21 was decreased (Figure 5C). Consequently, MITF overexpression significantly increased cell migration (Figure 5D) and invasion (Figure 5E). To determine whether RhoA/YAP signaling is dependent on MITF, we performed Western blot analysis and migration and invasion assays using siRNA against RhoA or YAP in MITF-overexpressing cells. Knockdown of RhoA significantly reduced the expression of YAP as well as cell migration and invasion. In addition, knockdown of YAP did not affect the expression of RhoA, but cell migration and invasion were decreased, reversing the effect of MITF overexpression. These results suggest that RhoA is upstream of YAP and that MITF is dependent on RhoA/YAP signaling (Figure S6). These results indicate that MITF promotes cell motility and invasiveness through activation of the canonical RhoA/YAP signaling pathway.

### 3.6. MITF Affects Tumorigenesis in ccRCC Cells In Vivo

**Figure 6:**
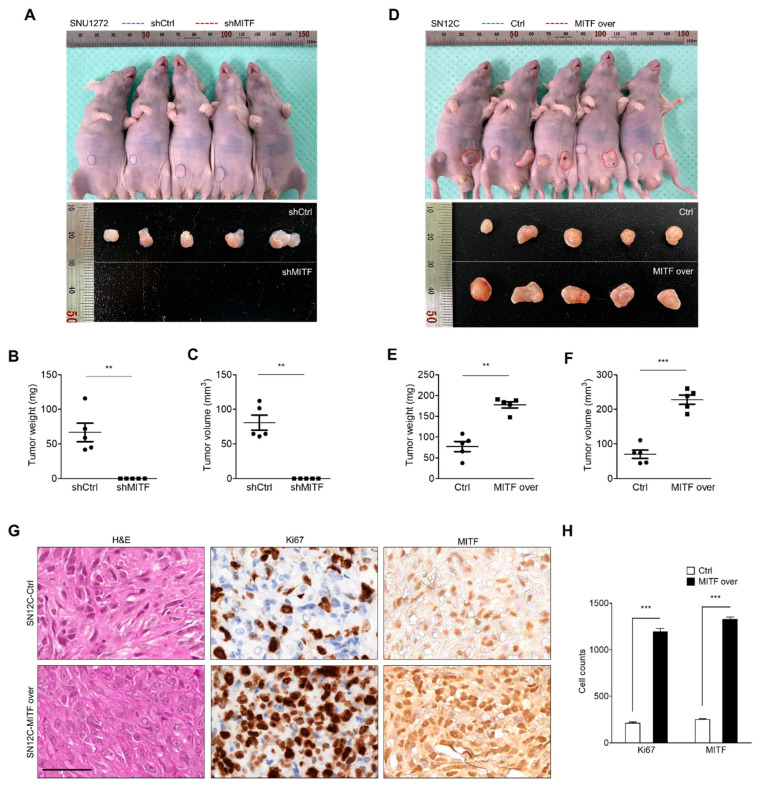
MITF affects tumorigenesis in ccRCC in vivo. (**A**) SNU1272-shCtrl and SNU1272-shMITF cells were subcutaneously injected into both flanks of BALB/c nude mice (*n* = 5). Upper panel: representative images showing tumor-bearing mice and sizes of tumors injected with SNU1272-shCtrl and SNU1272-shMITF cells at 60 days. Lower panel: representative images of the removed tumors. (**B**,**C**) Tumor weight and volume, evaluated after dissecting tumors from the mice in each group. Tumor growth was significantly impaired in the MITF knockdown group. Data are presented as the means ± standard deviation and were evaluated using Student’s t-test (*n* = 5). (**D**) SN12C-Ctrl and SN12C-MITF cells were subcutaneously injected into both flanks of BALB/c nude mice (*n* = 5). Upper panel: representative images showing tumor-bearing mice and the sizes of the tumors injected with SN12C-Ctrl and SN12C-MITF cells at 34 days. Lower panel: representative images of the removed tumors. (**E**,**F**) Tumor weight and volume, evaluated after dissecting tumors from the mice in each group. Tumor growth significantly increased in the MITF overexpression group. Data are presented as the means ± standard deviation and were evaluated using Student’s *t*-test (*n* = 5). (**G**,**H**) Representative images of H&E, Ki-67 and MITF staining (×400), and the numbers of Ki-67- and MITF-expressing cells. Data are presented as the means ± standard deviation and were evaluated using Student’s *t*-test (*n* = 5). Scale bars, 50 μm. **, *p* < 0.01; ***, *p* < 0.001.

We examined whether MITF knockdown and MITF overexpression affect tumor growth in vivo. SNU1272-shCtrl and SNU1272-shMITF cells were subcutaneously injected into nude mice and tumors were analyzed at 60 days post-injection. MITF-knockdown tumors were not observed (Figure 6A–C). Next, SN12C-Ctrl and SN12C-MITF-overexpressing cells were subcutaneously injected into nude mice and tumors were measured at 34 days post-injection. The tumors in the MITF-overexpression group were larger compared with controls (Figure 6D) in terms of volume and weight (Figure 6E,F). Immunohistochemistry (IHC) staining revealed that the expression of MITF and KI-67, a marker of cell proliferation, had increased in the tumor tissue derived from MITF overexpression cells, compared with the control (Figure 6G,H). These data suggest that MITF regulated tumor growth in nude mice. These in vivo data validate our in vitro results demonstrating that MITF contributes to ccRCC tumorigenesis as an oncogene.

## 4. Discussion

ccRCC is a cancer characterized by its high invasiveness, mortality and resistance to chemotherapy and radiation [3]. As there are a lack of biomarkers for early diagnosis, most ccRCC patients are diagnosed at a later stage, resulting in a relatively poor prognosis [27,28]. A recent study showed that renal carcinoma is associated with MITF/TFE translocation during the later stages of disease, i.e., when there is lymph node involvement [29,30]. MITF genetically predisposes patients to co-occurring melanoma and RCC [31]. In our study, we demonstrated that MITF is involved in ccRCC progression through activation of the RhoA/YAP signaling pathway. Moreover, we showed that MITF promoted cell motility, migration and invasion in vitro, and induced tumor formation in vivo in xenograft models of ccRCC.

MITF promotes the survival, proliferation and differentiation of melanocytes, and is involved in cancer progression [32]. MITF is overexpressed in hepatocellular and bile duct carcinoma, and has been associated with poor prognosis in hepatocellular carcinoma patients [20]. In pancreatic ductal adenocarcinoma, MITF has been identified as a master regulator of metabolic reprogramming via control of the autophagy-lysosome system [33]. Similarly, studies have shown that MITF is involved in autophagy and cellular homeostasis in lung cancer [22]. MITF plays an important role in cell division, induces differentiation-related cell cycle arrest, and promotes proliferation [34,35]. In the cell cycle, MITF exerts opposing and context-dependent actions through direct regulation of CDK2 and p21Cip1 [36,37]. In our study, knockdown of MITF significantly modulated cell growth by shifting the cell cycle from the G0/G1 phase to the S and G2/M phases in ccRCC. Notably, MITF is significantly associated with induction of the cell cycle-regulating proteins p21, CDK1, CDK2, cyclin A, cyclin B1 and cyclin E. In addition, MITF promotes cell proliferation, migration and invasion through YAP signaling.

YAP has been reported as an oncogene in a number of cancers. YAP is an effector of the evolutionarily conserved Hippo signaling pathway, and plays important roles in regulating cellular proliferation, survival and differentiation, as well as migration and invasion [38,39]. Overexpression of YAP protein and its nuclear localization were observed in colon, lung, pancreatic, hepatocellular, ovarian, and prostate carcinomas [40,41,42]. YAP/Hippo signaling promotes the progression of triple negative breast cancer in conjunction with members of the AP1 family, and is associated with poor survival [43,44]. The mechanism underlying the expression and activity of YAP in ccRCC is not well known. The present study demonstrated that MITF knockdown inhibited YAP translocation from the cytoplasm to the nucleus. In addition, overexpression of YAP enhanced cell migration and cellular invasion, whereas these effects were reversed by MITF knockdown. To the best of our knowledge, this study is the first to implicate YAP signaling, via MITF, in increased invasiveness and metastatic potential in ccRCC. YAP transcriptional activity is regulated by G-protein binding receptor (GPCR)-mediated extracellular signaling via Rho GTPase or F-actin [45,46].

As a major member of the Rho guanosine triphosphatase (GTPase) family, RhoA is involved in tissue-specific cytoskeleton dynamics, actin stress fiber formation, cell focal adhesion, survival, cell cycle progression and transcription regulation [47,48]. RhoA is critical for the migration, invasion and metastasis of various cancers, including RCC [49,50]. However, little is known about the role of RhoA in ccRCC. In our study, we showed that MITF regulates RhoA, Rac1,2,3 and Cdc42. In addition, we found that phosphorylation of FAK and YAP expression levels were increased, while phosphorylated YAP was decreased, by CN03. Moreover, YAP was significantly associated with the induction of cell cycle-related proteins through RhoA activation. These results indicate that MITF overexpression upregulates cell motility and invasiveness through the canonical RhoA/YAP signaling pathway.

CRIK is a downstream substrate of the Rho protein. During cellular contraction, CRIK binds to RhoA to enhance and maintain contractile ring dynamics [51,52]. CRIK has been linked to the development of cancer in humans. In colon cancer, the expression of CRIK is increased, and CRIK knockdown reduces cancer cell proliferation through the p53 signaling pathway [26]. Similarly, in breast cancer, CRIK promotes cancer aggressiveness and tumorigenesis [53]. In our study, the expression of CRIK was shown to be regulated by MITF. However, the association between MITF and CRIK in ccRCC is not yet clear. Future studies are necessary to understand the molecular mechanisms underlying the activities of MITF and CRIK in ccRCC.

## 5. Conclusions

In this study, we demonstrated that MITF plays an important role in the progression of ccRCC. MITF was significantly associated with tumor progression in ccRCC, both in vitro and in vivo. MITF is involved in RhoA/YAP signaling-mediated cell proliferation, migration and invasion in ccRCC. More importantly, we demonstrated that MITF regulates tumor progression in ccRCC cell via the RhoA/YAP signaling pathway; taken together, the results suggest that MITF could be a potential therapeutic target in ccRCC.

## Data Availability

The data presented in this study are contained within the article and the Supplementary Materials.

## Supplementary Materials

The following are available online at https://www.mdpi.com/article/10.3390/cancers13122920/s1, Figure S1: Immunofluorescence of MITF in MITF-knockdown and shCtrl cells, Figure S2: Cell cycle analysis by FACS showed an increase in the populations of MITF-knockdown cells in the S and G2/M phases after propidium iodide (PI) staining, Figure S3: Immunofluorescence staining showing that MITF knockdown reduced phalloidin expression, Figure S4: The relationship of MITF expression and YAP expression in clear cell renal cell carcinoma, Figure S5: YAP overexpression was determined by immunofluorescence staining, Figure S6: RhoA/YAP signaling is MITF-dependent, Figure S7: Uncropped blots.
