# Supplementary material for: MITF Promotes Cell Growth, Migration and Invasion in Clear Cell Renal Cell Carcinoma by Activating the RhoA/YAP Signal Pathway
Source: Cancers (Basel). 2021 Jun 11;13(12):2920. doi: 10.3390/cancers13122920 (PMC8230652; doi:10.3390/cancers13122920)
Supplement: Supplementary file 1 [file cancers-13-02920-s001.zip › cancers-1228272-supplementary.pdf]

Supplementary Materials

MITF Promotes Cell Growth, Migration and Invasion in Clear Cell Renal Cell Carcinoma by Activating the RhoA/YAP Signal Pathway

Nayoung Kim, Solbi Kim, Myung-Won Lee, Heung-Jin Jeon, Hyewon Ryu, Jin-Man Kim and Hyo-Jin Lee

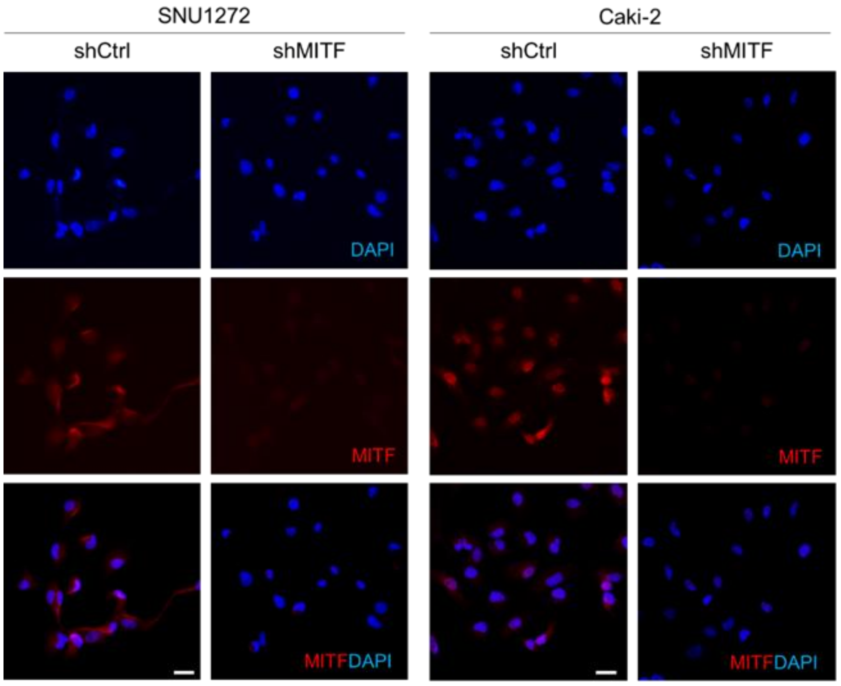

Figure S1. Immunofluorescence of MITF in MITF-knockdown and shCtrl cells. Scale bars, 20 μm.

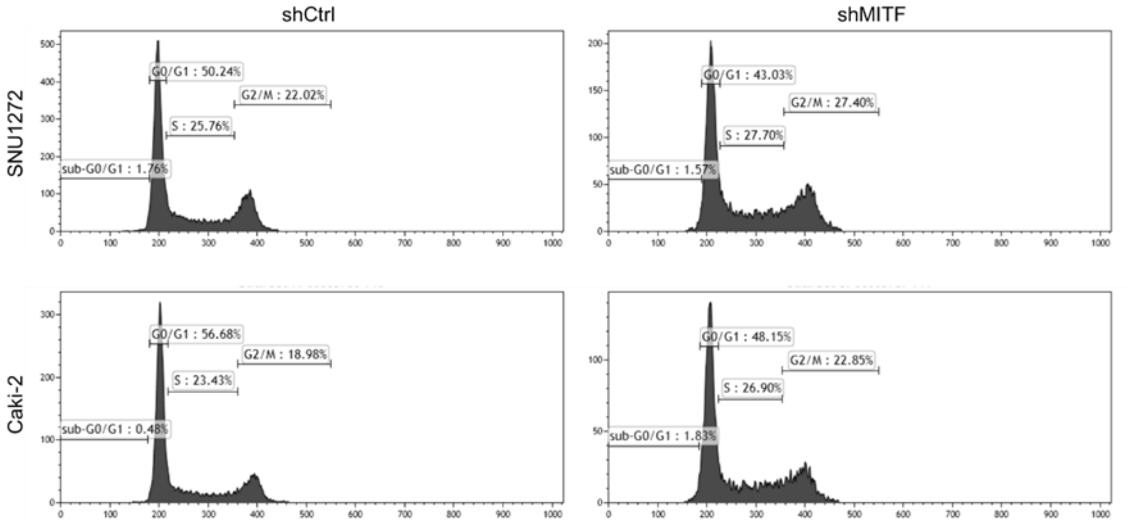

Figure S2. Cell cycle analysis by FACS showed an increase in the populations of MITF-knockdown cells in the S and G2/M phases after propidium iodide (PI) staining.

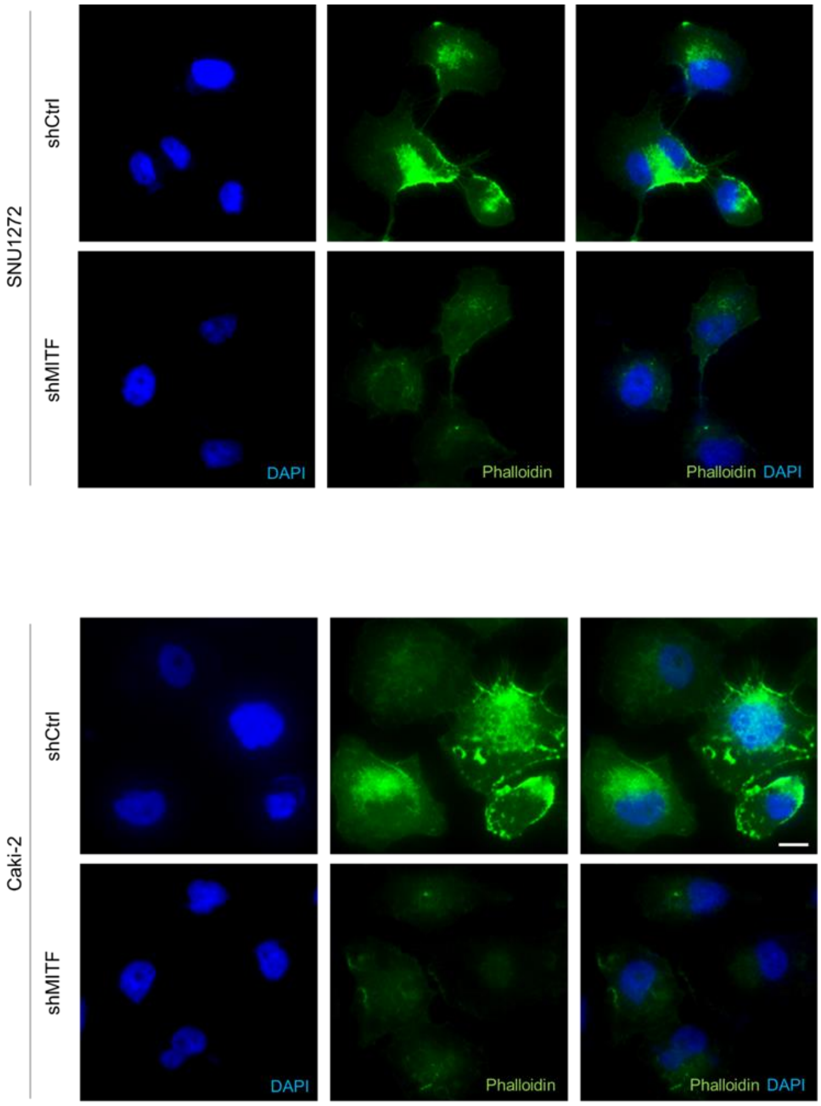

Figure S3. Immunofluorescence staining showing that MITF knockdown reduced phalloidin expression. Scale bars, 10 μm.

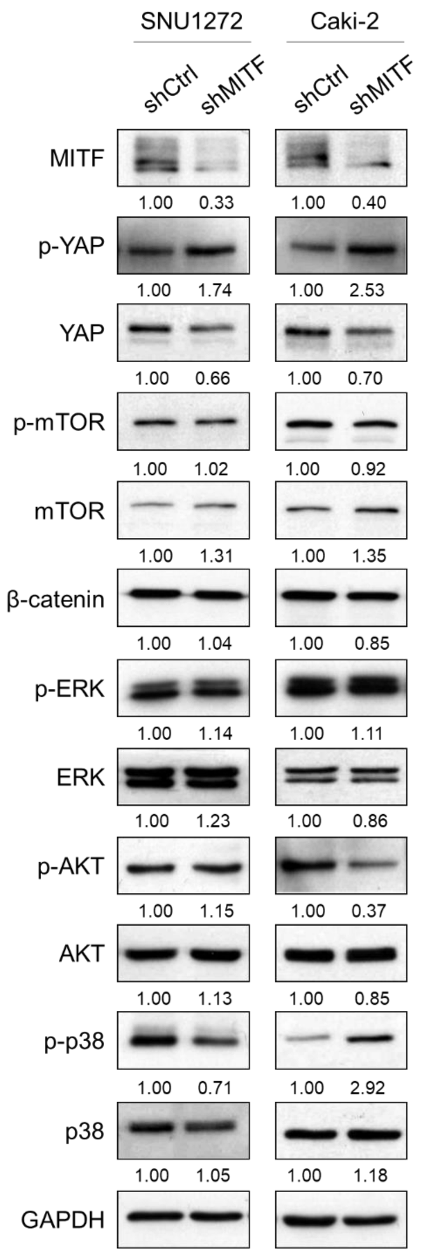

Figure S4. The relationship of MITF expression and YAP expression in clear cell renal cell carcinoma. Western blotting of several cancer-related pathways. Whole areas were measured using ImageJ software (*n* = 3). Uncropped blots are shown in Figure S7.

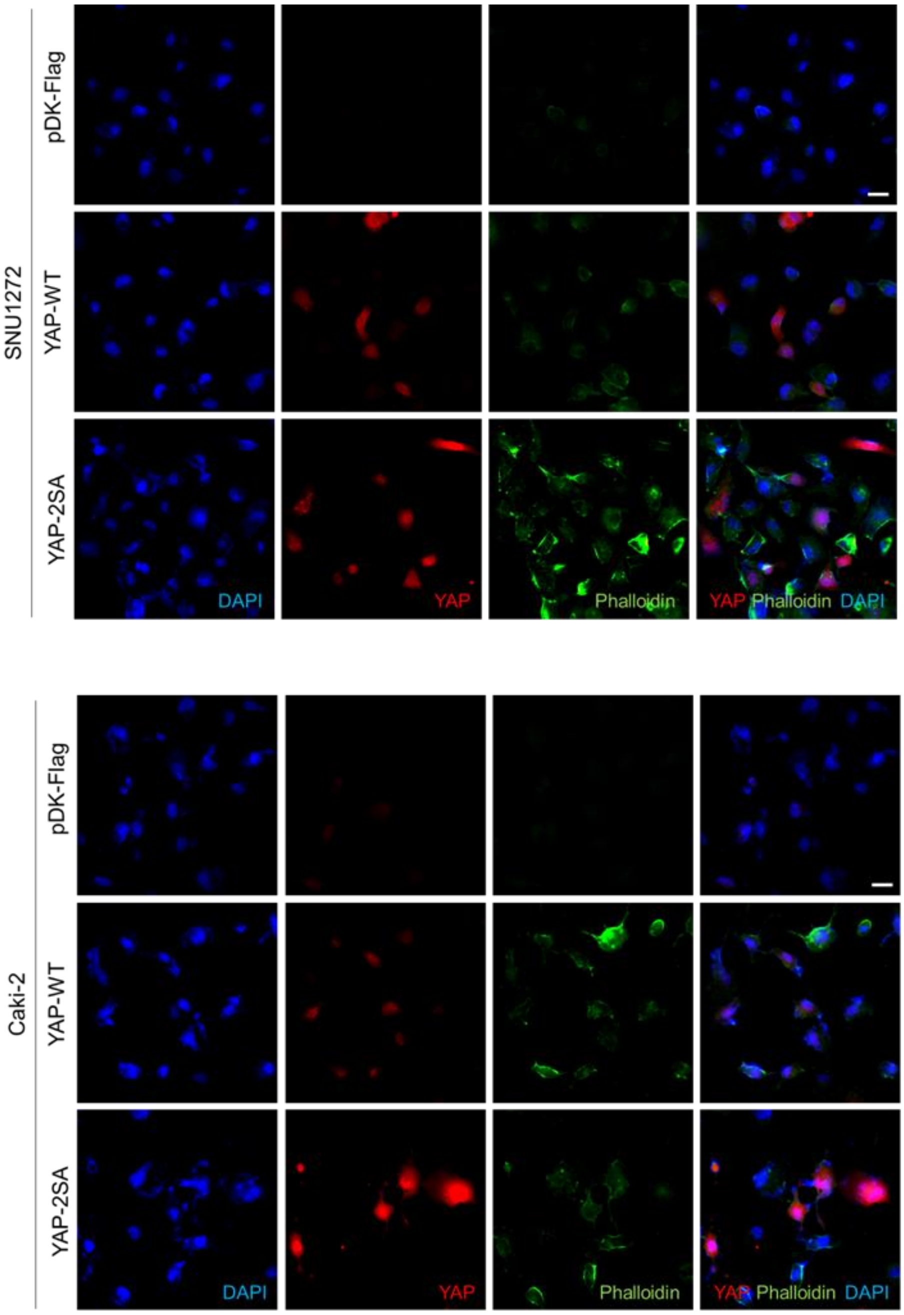

Figure S5. YAP overexpression was determined by immunofluorescence staining. Scale bars, 20 μm.

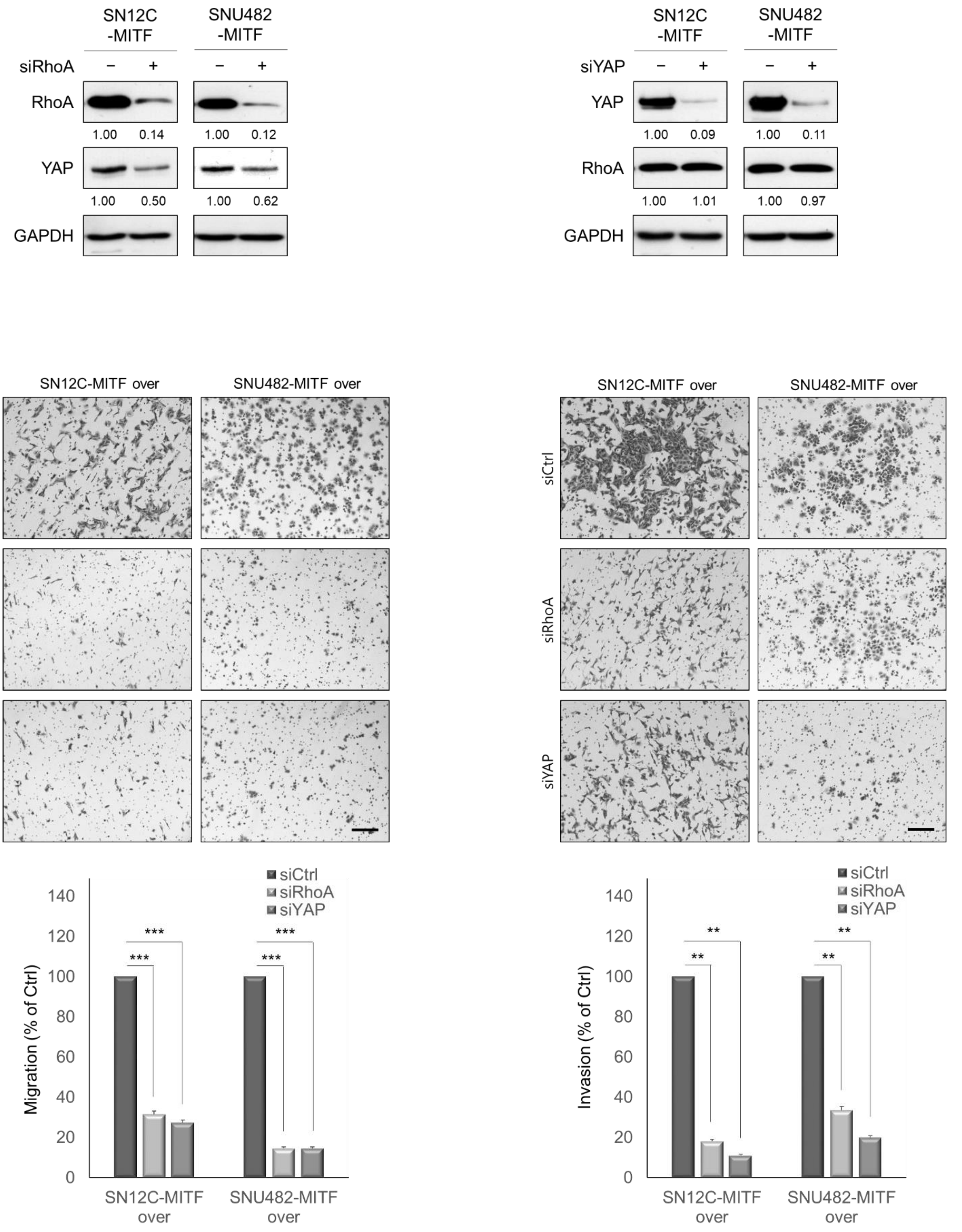

Figure S6. RhoA/YAP signaling is MITF-dependent. Knockdown of RhoA significantly reduced the expression of YAP, but knockdown of YPA did not affect the expression of RhoA. Whole areas were measured using ImageJ software (*n* = 3). Uncropped blots are shown in Figure S7. Transwell migration and invasion assays showing that knockdown of RhoA or YAP reduced MITF overexpression-induced cell migration and invasion. The number of cells in five randomly chosen fields was counted. Data are presented as the means ± standard deviation and were evaluated using Student's *t*-test (*n* = 3). Scale bar, 200 μm. **, $p < 0.01$; ***, $p < 0.001$.

Un-cropped images for Figure 1.

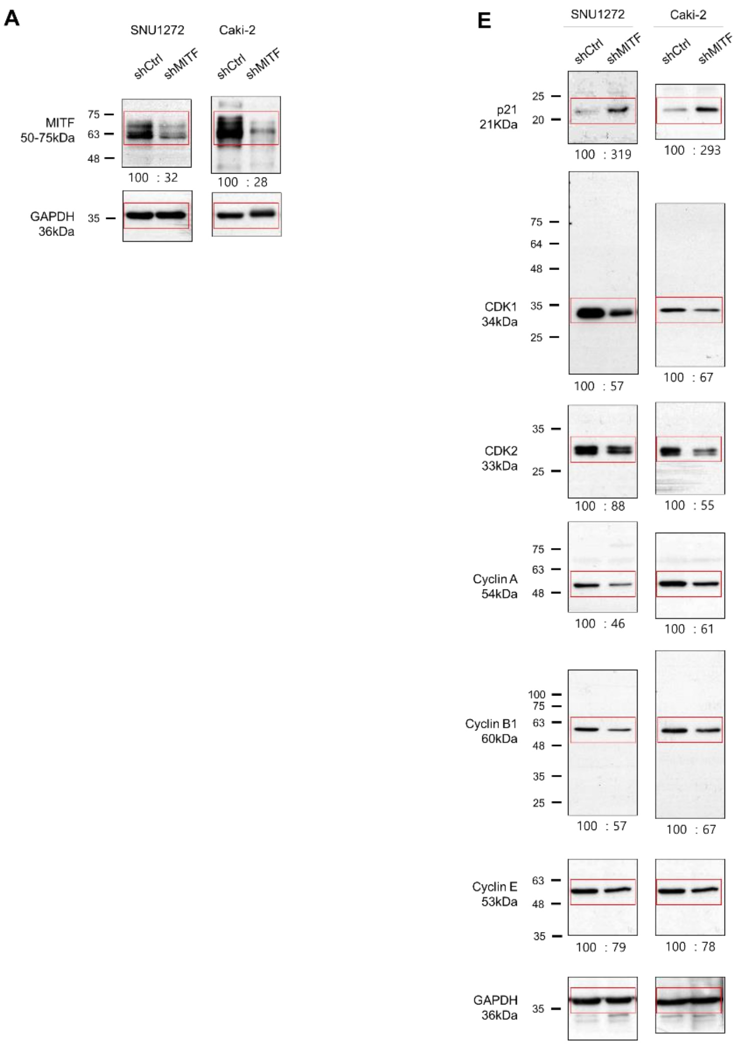

Un-cropped images for Figure 2.

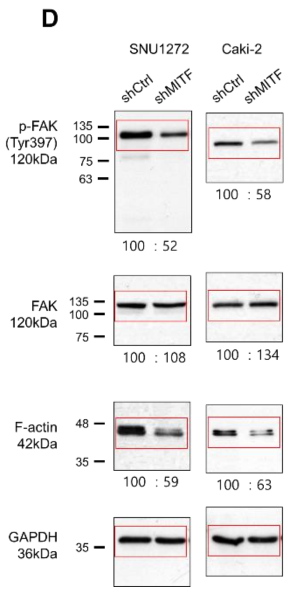

Un-cropped images for Figure 3.

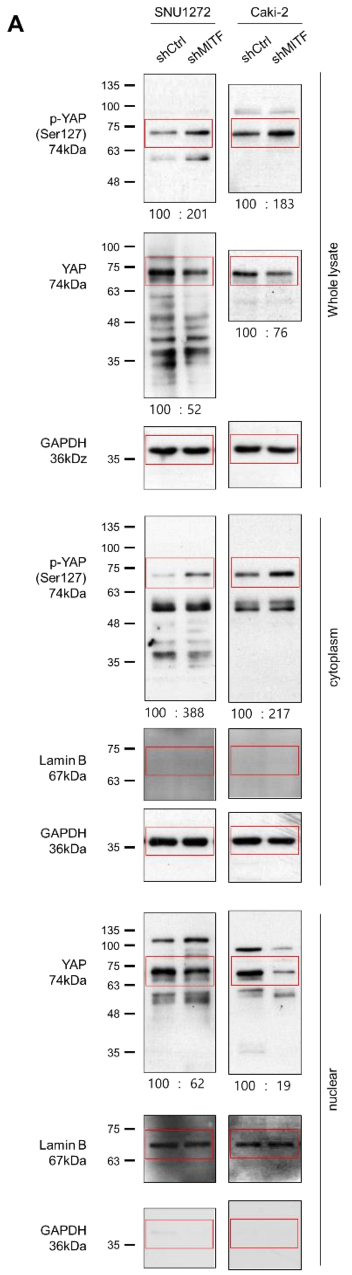

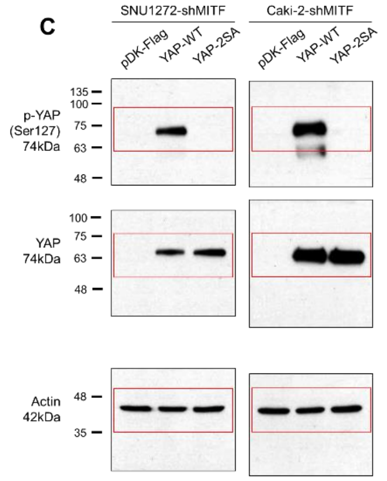

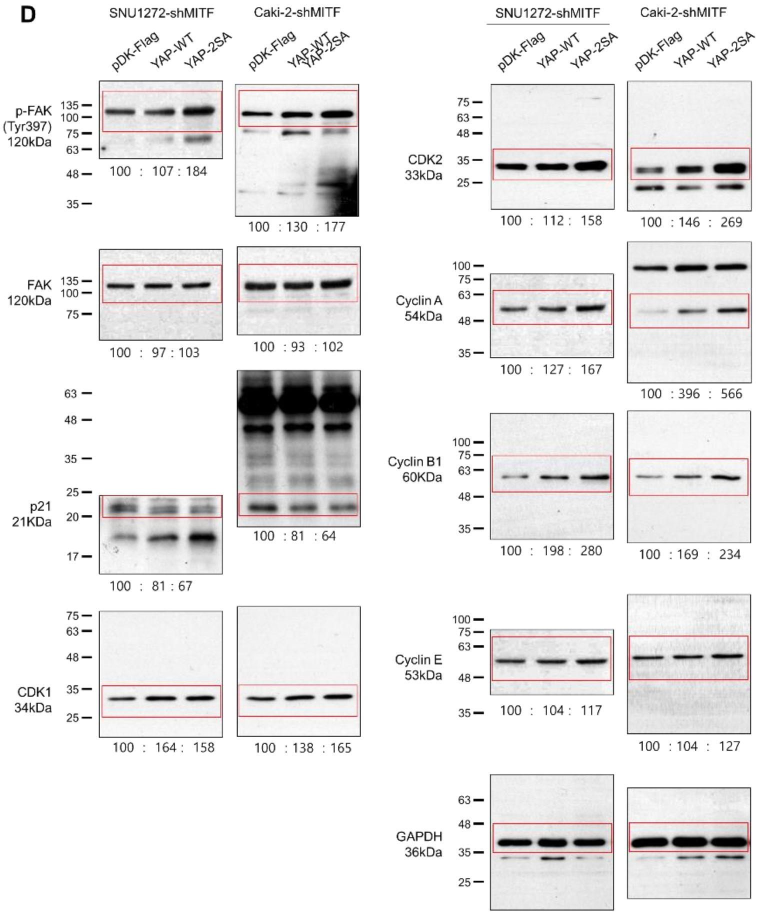

Un-cropped images for Figure 4.

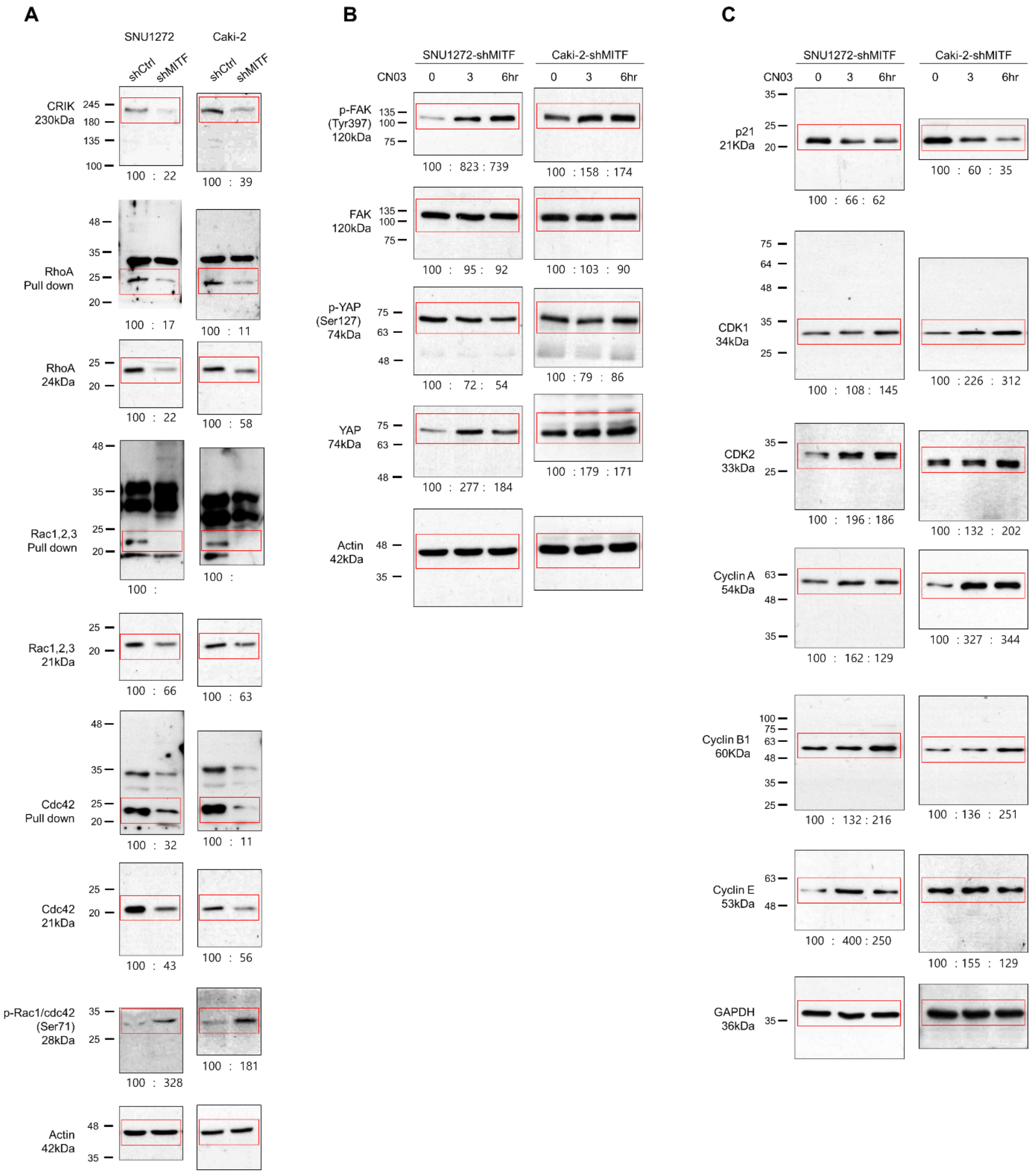

Un-cropped images for Figure 5.

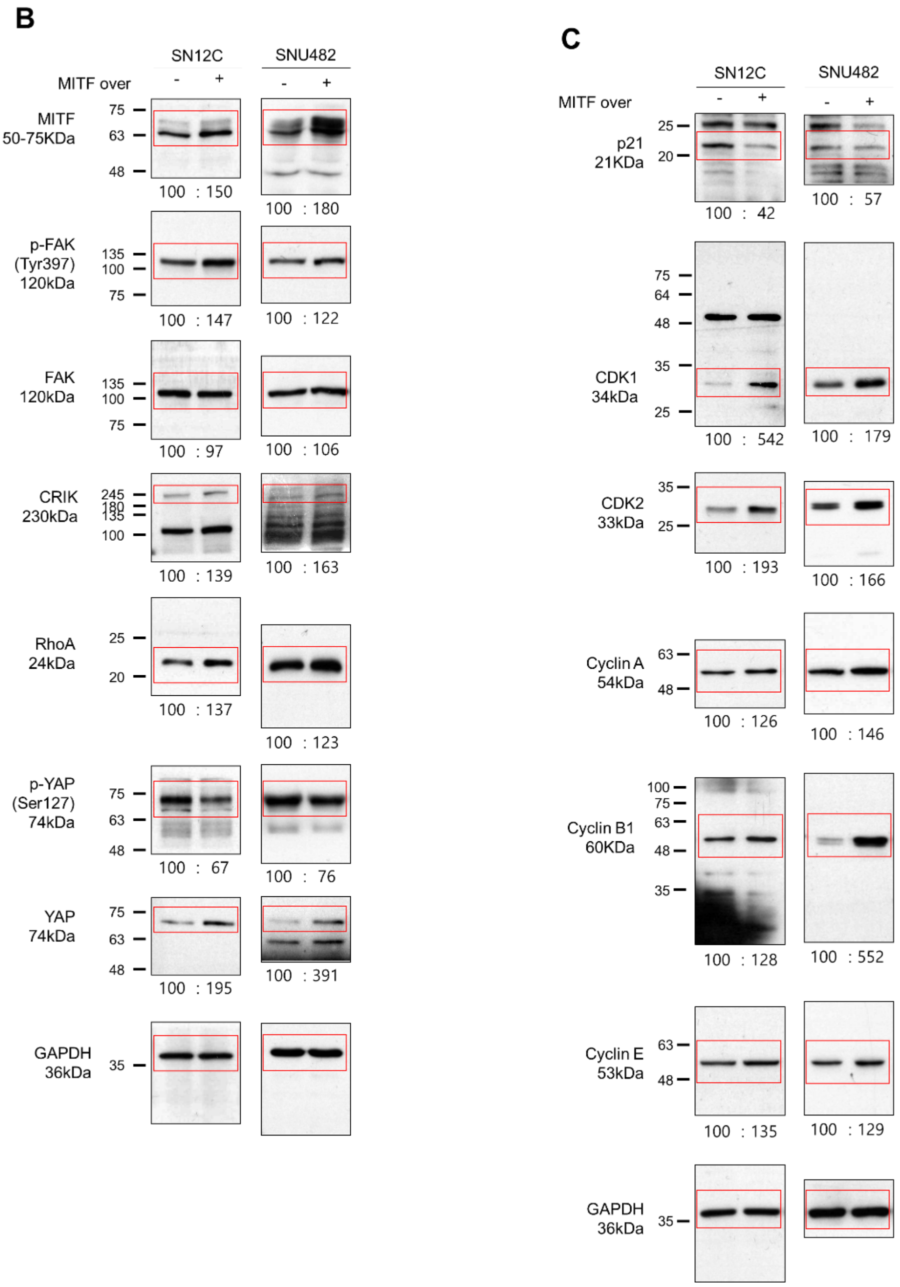

Un-cropped images for Figure S4.

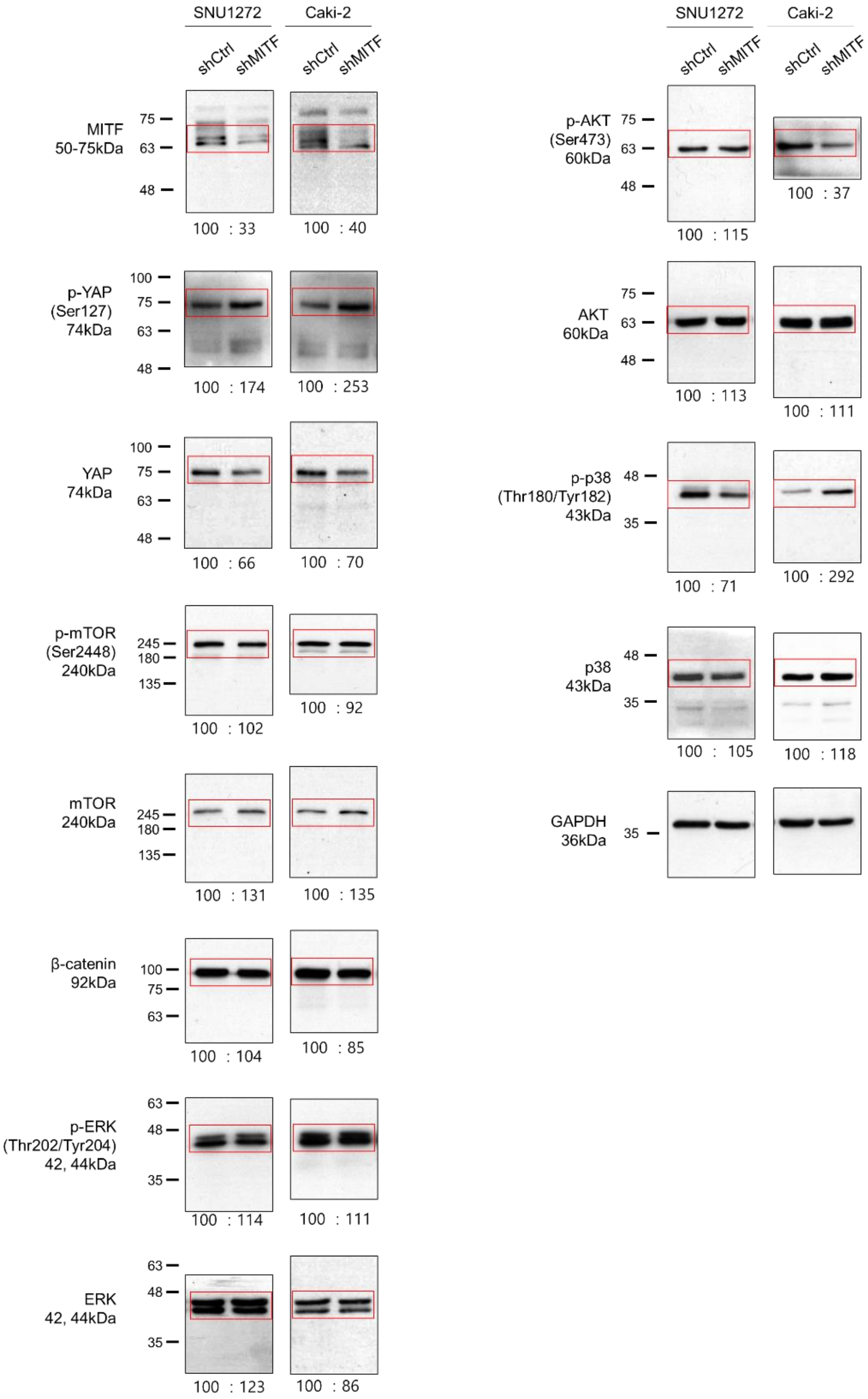

Un-cropped images for Figure S6.

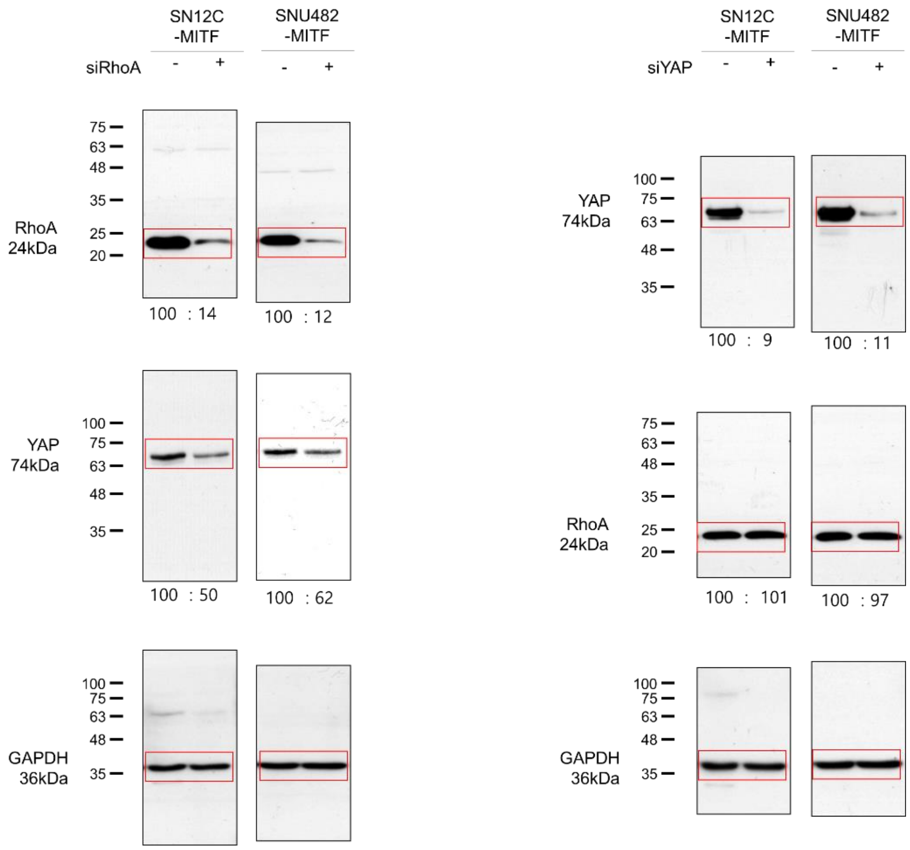

Figure S7. Un-cropped blots.
